# Chemotherapie ± Androgenrezeptorantagonisten beim metastasierten hormonsensitiven Prostatakarzinom

**DOI:** 10.1007/s00120-023-02029-0

**Published:** 2023-02-10

**Authors:** Mike Wenzel, Benedikt Hoeh, Felix K. H. Chun, Philipp Mandel

**Affiliations:** Klinik für Urologie, Universitätsklinikum Frankfurt, Theodor-Stern-Kai 7, 60590 Frankfurt, Deutschland

**Keywords:** Tripple-Therapie, Kombinationstherapie, Abirateron, Docetaxel, Metastasen, mHSPC, Darolutamide, Triplet therapy, Combination therapy, Abiraterone, Docetaxel, Metastasis, neoplasm, mHSPC, Darolutamide

## Abstract

**Hintergrund:**

Nachdem die alleinige Androgendeprivationstherapie (ADT) lange Zeit die Goldstandardbehandlung des metastasierten hormonsensitiven Prostatakarzinoms (mHSPC) war, wurde diese in den letzten Jahren durch Doublet-Kombinationstherapien aus ADT + erweiterte Hormontherapie (ARTA, „androgen receptor targeted agent“) oder ADT + Docetaxel-Chemotherapie abgelöst. Erstmals stehen nun Daten aus Triplet-Kombinationstherapien aus ADT + ARTA (Abirateron/Darolutamid) + Docetaxel-Chemotherapie zur Verfügung.

**Fragestellung:**

Welcher mHSPC-Patient profitiert von einer „Doublet“- vs. „Triplet-Kombinationstherapie“ und welches Nebenwirkungsspektrum ist jeweils zu erwarten?

**Ergebnisse:**

Die aktuellen Triplet-Therapien (ADT + Docetaxel + Abirateron/Darolutamid) zeigen eine Verlängerung des Gesamtüberlebens gegenüber der Doublet-Therapie aus ADT + Docetaxel aller mHSPC (ARASENS) bzw. primär metastasierten „High-volume-“ (PEACE-1) mHSPC-Patienten. Im Setting des High-volume-mHSPC zeigt sich dieser positive Gesamtüberlebenseffekt explizit für die Triplet-Kombination aus ADT + Docetaxel + Abirateron. Beim Low-volume-mHSPC zeigt sich dieser Effekt lediglich für das progressionsfreie Überleben – jedoch nicht für das Gesamtüberleben. Ähnliche Darolutamid‑/Triplet-Kombinationstherapie’ Daten (High- vs. Low-volume-mHSPC) liegen aktuell nicht vor. Die Nebenwirkungsraten von „Triplet- vs. Doublet-Kombinationstherapie“ sind nur leicht erhöht und v. a. auf typische Chemotherapie-assoziierte (Neutropenie) und Androgenrezeptorantagonisten (ARTA)-spezifische Nebenwirkungen (Abirateron) zurückzuführen.

**Zusammenfassung:**

Die ADT-Mono- und die „Doublet-Kombinationstherapie“ aus ADT + Docetaxel sollten in der Erstlinientherapie beim mHSPC keine Rolle mehr spielen. Bis zum Vorliegen weiterführender Daten über den Zusatznutzen der „Triplet-Kombinationstherapie“ in relevanten Subgruppen, stellen die Kombinationstherapien aus ADT + ARTA bzw. ADT + ARTA + Docetaxel in Abhängigkeit patientenspezifischer Charakteristika (Alter, ECOG [Eastern Cooperative Oncology Group], Metastasenlast, primäre/sekundäre Metastasierung) die aktuelle primären Therapieoptionen dar.

## Abstract (en)

**Background:**

Although androgen deprivation therapy (ADT) alone has been the standard of care (SOC) in the treatment of metastatic hormone-sensitive prostate cancer (mHSPC) for decades, combination therapies of novel hormone therapy (androgen receptor-targeting agents [ARTA]) or docetaxel chemotherapy have more recently replaced single ADT treatment. In addition, data for triplet therapies with ADT plus ARTA (abiraterone/darolutamide) and docetaxel chemotherapy are now available.

**Objectives:**

The present review addresses the question which therapy is suitable for which mHSPC patient. Who benefits from doublet therapy and which patient from triplet therapy? Which side effects can be expected?

**Results:**

Triplet therapy consisting of ADT + docetaxel + abiraterone/darolutamide resulted in a significantly longer overall survival compared to therapy consisting of ADT + docetaxel of all mHSPC (ARASENS) and primary metastatic high-volume (PEACE-1) mHSPC patients. In the setting of high-volume mHSPC, prolonged overall survival is seen for the specific triplet combination of ADT + docetaxel + abiraterone. In the low-volume mHSPC setting, only an extended progression-free survival but not overall survival was observed. Data regarding the classification of high- vs. low-volume mHSPC for the triplet therapy consisting of darolutamide are currently not available. Side effects with triplet therapies are almost comparable with those of doublet therapies and relate to typical chemotherapy-associated (neutropenia) and ARTA-specific side effects (abiraterone).

**Conclusion:**

ADT alone or ADT + docetaxel should no longer play a role in first-line therapy for mHSPC. Accordingly, therapy consisting of ADT + ARTA or ADT + ARTA + docetaxel represents the current primary treatment option pending further data and regarding patient-specific characteristics (age, ECOG status, metastatic burden, primary/secondary metastatic disease).

## Einleitung

Über mehrere Dekaden hinweg war die alleinige Androgendeprivationstherapie (ADT) der Goldstandard („standard of care“, SOC) in der Behandlung von Patienten mit metastasiertem hormonsensitivem Prostatakarzinom (mHSPC). Nachdem im Jahr 2004 erstmalig eine Kombinationstherapie aus ADT plus Docetaxel im Stadium des metastasierten kastrationsresistenten Prostatakarzinoms (mCRPC) einen (vergleichsweise kurzen) Überlebensvorteil in der TAX-327-Studie zeigte, verschob sich der Fokus der Forschung in den letzten 10 Jahren vermehrt auf den Bereich des mHSPC [1]. Innerhalb weniger Jahre zeigten Kombinationstherapien einen signifikanten Überlebensvorteil mit einer Kombination aus ADT plus Docetaxel-Chemotherapie oder neueren Androgenrezeptorantagonisten (ARTA, „androgen receptor targeted agent“, ARTA) wie Abirateron bei zunächst spezifischen Metastasierungsmustern („high volume“ nach CHAARTED oder „high risk“ nach LATITUDE; [2, 3]). Weitergehend in der Forschung konnte dann erstmalig 2018 bzw. 2019 die ARTA-Kombinationstherapie (Enzalutamid und Apalutamid) zusätzlich zur ADT einen signifikanten Überlebensvorteil unabhängig von der Metastasenlast und dem Zeitpunkt der Metastasierung (primäres vs. sekundäres mHSPC) bestätigen [4–6].

Erstmalig konnten die auf dem ASCO 2021 (American Society of Clinical Oncology) vorgestellten Daten zur sog. Triplet-Therapie aus ADT plus Docetaxel und Abirateron einen signifikanten Überlebensvorteil im Vergleich zum SOC aus ADT und Docetaxel nachweisen (PEACE‑1; [7]). Simultane Ergebnisse zeigte die 2022 publizierte ARASENS-Studie aus ADT plus Docetaxel und Darolutamid [8]. Bisweilen vergeht nun kaum ein internationaler Kongress, an dem nicht Daten hinsichtlich neuer 2‑ bzw. 3‑Fach-Kombinationstherapien für das mHSPC oder mCRPC vorgestellt werden.

**Figure 1:**
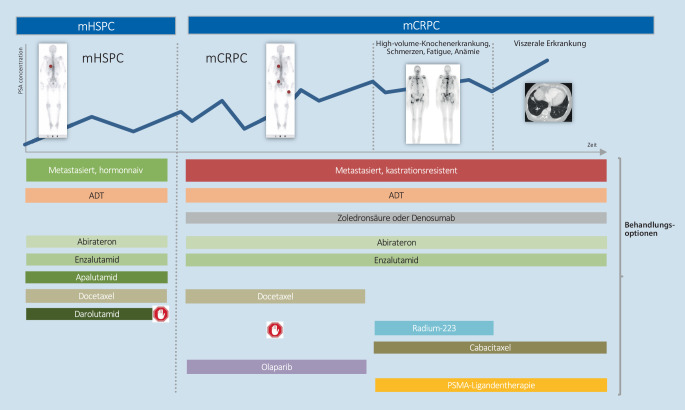

Zusammenfassend hat das Feld der medikamentösen Behandlung des mHSPC einen grundlegenden Wandel innerhalb der letzten 10 Jahre durchlebt, der sehr wahrscheinlich noch nicht am Ende ist. Urologen und Urologinnen aller Länder stehen nun vor der Frage und der Qual der Wahl: Doublet- vs. Triplet-Therapie beim mHSPC und für welchen Patienten (Abb. 1)? Die vorliegende Übersichtsarbeit hat sich somit zum Ziel gesetzt, die unterschiedlichen Therapieoptionen in Abhängigkeit der Vor- und Nachteile zu beleuchten. Denn z. T. steckt hier der Teufel im Detail.

## Direkter und indirekter Vergleich der Kombinationstherapien

Wie in Abb. 1 dargestellt, gibt es im Stadium des mHSPC aktuell vier in Deutschland zugelassene Kombinationstherapien aus ADT + ARTA („androgen receptor targeted agent“; Abirateron, Enzalutamid oder Apalutamid) sowie die ADT + Docetaxel-Chemotherapie. Alle Substanzen konnten in den aktuellsten publizierten Daten bezüglich der Endpunkte des Gesamtüberlebens (OS), als auch des progressionsfreien Überlebens (PFS) signifikante Vorteile der Doublet-Therapien gegenüber der alleinigen ADT in der Gesamtkohorte (unabhängig der Metastasenlast) darstellen (Tab. 1, Hazard Ratio [HR] Range zwischen 0,61–0,88).

**Table 1:** 

–	ENZAMET	CHAARTED	GETUG-AFU15	STAMPEDE ARM C	LATITUDE
*Autor, Einschlusszeitraum*	Davis et al. 2014–2017 [4]	Kyriakopoulos et al. 2006–2012 [2]	Gravis et al. 2004–2008 [22]	Clarke et al. 2005–2013 [20]	Fizazi et al. 2012–2014 [3]
*Kontroll- vs. Verumarm*	ADT vs. Enzalutamid & ADT	ADT vs. Docetaxel & ADT	ADT vs. Docetaxel & ADT	ADT vs. Docetaxel & ADT	ADT vs. Abirateron & ADT
*Einschlusskriterien*	mHSPC, bis zu 2 Zyklen Chemotherapie vorab	mHSPC, keine Chemotherapie vorab	mHSPC, keine Chemotherapie vorab	mHSPC, keine Chemotherapie vorab	mHSPC, 2 von 3 High-risk-Kriterien^a^
*Metastasierungsmuster*	Synchron + metachron	Synchron + metachron	Synchron + metachron	Synchron	Synchron
*Performance status*	ECOG 0–2	ECOG 0–2	ECOG 0–2	WHO 0–2	ECOG 0–2
*Patientenanzahl* Kontrolle/Verum	562/563	393/397	193/192	724/362	597/602
*Medianes Follow-up* (Monate)	34	54	84	78	52
*Medianer PSA (IQR;* ng/ml) Kontrolle/Verum	–	52,1 (0,1–8056) vs.50,9 (0,2–8540)	59 vs. 55	102,5 (32,8–354) vs. 97 (40,5–340)	–
*Gleason-Score Anteil* *≥* *8* (%) Kontrolle/Verum	57 vs. 60	62 vs. 61	24 vs. 32	66 vs. 70	97 vs. 98
*Anteil Knochenmetastasen* (%) Kontrolle/Verum	82 vs. 80	–	81 vs. 81	88 vs. 85	98 vs. 97
*Anteil viszeraler Metastasen* (%) Kontrolle/Verum	12 vs. 11	17 vs. 14	12 vs. 15	13 vs. 13	22 vs. 22
„*High volume disease*“ (%) *Kontrolle/Verum*	52 vs. 53	64 vs. 66	47 vs. 48	44 vs. 41	78 vs. 82
*Anteil vorherige Chemotherapie* (%) Kontrolle/Verum	16,9 vs. 14,8	0 vs. 0	0 vs. 0	0 vs. 0	0 vs. 0
*Anteil vorherige Lokaltherapie* (%) Kontrolle/Verum	42 vs. 42	27 vs. 27	24 vs. 32	5 vs. 4	3 vs. 4
*Gesamtüberleben *(HR; 95 %-KI) Kontrolle/Verum	0,67 (0,52–0,86)	0,72 (0,59–0,89)	0,88 (0,68–1,14)	0,81 (0,69–0,95)	0,66 (0,56–0,78)
*Gesamtüberlebem (*„*low volume*“*,* HR; 95 %-KI) Kontrolle/Verum	0,43 (0,26–0,72)	1,04 (0,7–1,55)	1,02 (0,67–1,55)	0,76 (0,54–1,07)	0,72 (0,47–1,10)
*Gesamtüberleben (*„*high volume*“*,* HR: 95 %-KI) Kontrolle/Verum	0,8 (0,59–1,07)	0,63 (0,5–0,79)	0,78 (0,56–1,09)	0,81 (0,64–1,02)	0,62 (0,52–0,74)
–	**STAMPEDE-ARM G**	**TITAN**	**ARCHES**	**PEACE‑1**	**ARASENS**
*Autor, Einschlusszeitraum*	James et al. 2011–2014 [19]	Chi et al. 2015–2017 [6]	Armstrong et al. 2016–2018 [5]	Fizazi et al. 2013–2018 [7]	Smith et al. 2016–2018 [8]
*Kontroll- vs. Verumarm*	ADT vs. Abirateron & ADT	ADT vs. Apalutamid & ADT	ADT vs. Enzalutamid & ADT	Docetaxel & ADT (±RT) vs. Abiraterone & Docetaxel (±RT) & ADT	Docetaxel & ADT vs. Darolutamide & Docetaxel & ADT
*Einschlusskritrien*	mHSPC oder N1 oder 2 High-risk-Kriterien oder High-risk-Rezidiv^b^	mHSPC; mindestens eine Knochenmetastase	mHSPC	mHSPC	mHSPC
*Metastasierungsmuster*	Synchron + metachron	Synchron + metachron	Synchron + metachron	Synchron	Synchron + metachron
*Performance status*	WHO 0–2	ECOG 0–1	ECOG 0–1	ECOG 0–2	ECOG 0–1
*Patientenanzahl* Kontrolle/Verum	452/449	527/525	576/574	355/355	654/651
*Medianes Follow-up* (Monate)	73	44	45	36	43
*Medianes Alter (IQR;* Jahre) (Kontrolle/Verum)	67 (62–72) vs. 67 (62–71)	68 (43–90) vs. 69 (45–94)	70 (42–92) vs. 70 (46–92)	66 (59–70) vs. 66 (60–70)	67 (42–86) vs. 67 (41–89)
*Medianer PSA (IQR;* ng/ml) Kontrolle/Verum	97,2 (26–358) vs. 96,3 (29–371)	4 (0–2229) vs. 6 (0–2682)	5,1 (0–19000) vs. 5,4 (0–4832)	12 (3,0–59,9) vs. 13,7 (2,4–58,9)	24,2 (0–11947) vs. 30,3 (0–9219)
*Gleason-Score Anteil* *≥* *8* (%) Kontrolle/Verum	76 vs. 77	67 vs. 68	65 vs. 67	79 vs. 77	79 vs. 78
*Anteil Knochenmetastasen* (%) Kontrolle/Verum	65 vs. 66	100 vs. 100	75 vs. 75	79 vs. 81	80 vs. 79
*Anteil viszeraler Metastasen* (%) Kontrolle/Verum	0,4 vs. 0,2	14 vs. 11	11 vs. 11	12 vs. 13	18 vs. 17
„*High volume disease*“ (%) (Kontrolle/Verum)	57 vs. 54	64 vs. 62	64,8 vs. 61,7	65 vs. 63	–
*Anteil vorherige Chemotherapie* (%) Kontrolle/Verum	0 vs. 0	10 vs. 11	18 vs. 178	0 vs. 0	0 vs. 0
*Anteil vorherige Lokaltherapie* (%) Kontrolle/Verum	5 vs. 5	15 vs. 18	28 vs. 25	–	–
*Gesamtüberleben* (HR; 95 %-KI) Kontrolle/Verum	0,61 (0,49–0,79)	0,65 (0,53–0,79)	0,66 (0,53–0,81)	0,75 (0,59–0,95)	0,68 (0,57–0,80)
*Gesamtüberlebem (*„*low-volume*“, HR; 95 %-KI) Kontrolle/Verum	0,64 (0,42–0,97) [19]	0,52 (0,35–0,79)	0,66 (0,43–1,03)	0,83 (0,50–1,38)	–
*Gesamtüberleben (*„*high volume*“*, *HR; 95 %-KI) Kontrolle/Verum	0,60 (0,46–0,78) [19]	0,70 (0,56–0,88)	0,66 (0,52–0,83)	0,72 (0,55–0,95)	–

High-risk-Rezidiv nach Lokaltherapie: PSA > 4 ng/ml und Verdopplungszeit < 6 Monate oder PSA > 20 ng/ml; Lymphknoten oder Metastasenrezidiv oder < 12 Monate mit ADT und Intervall > 12 Monate ohne ADT bevor Einschluss

*mHSPC* metastasiertes hormonsensitives Prostatakarzinom, *ADT* Androgendeprivationstherapie, *IQR* Interquartilsabstand, *PSA* prostataspezifisches Antigen, *HR* Hazard Ratio, *WHO* World Health Organization, *ECOG* Eastern Cooperative Oncology Group, *KI* Konfidenzintervall

^a^Zwei der folgenden High-risk-Kriterien: Gleason-Score ≥ 8, ≥ 3 Knochemetastasen, ≥ 1 viszerale Metasase

^b^Schließt metastasierte und nicht-metastasierte Patienten ein. Kriterien für nicht-metastasierte: 1) 2 von folgender 3 Kriterien: cT3/T4 oder PSA > 40 ng/ml oder Gleason-Score 8–10. 2) Hochrisiko Kriterien für ein Rezidiv nach radikaler Prostatektomie oder Radtiotherapie: PSA > 4 ng/ml PSA-Verdopplungszeit < 6 Monate oder PSA > 20 ng/ml. 3) Lymphknoten oder Metastasenrezidiv oder < 12 Monate mit ADT und Intervall > 12 Monate ohne ADT vor Studieneinschluss

### Indirekter Vergleich der Doublet-Kombinationstherapie

Da ein direkter Vergleich zwischen den einzelnen ARTA-Substanzen nicht als klinische Phase-III-Studie zu erwarten ist, bedarf der Vergleich der Substanzen, sofern man diesen anstellen möchte, einen anderen statistischen Blickwinkel des indirekten Vergleichs: Bereits mehrere sog. Network-Metaanalysen haben die unterschiedlichen Therapieformen der Doublet-Kombinationstherapien im Vergleich zur alleinigen ADT verglichen: Beispielsweise konnten Mori et al. [9] zeigen, dass auch nach einem Pooling aller ADT-Kontrollgruppen, die Doublet-Kombinationstherapien weiterhin einen deutlichen Überlebensvorteil gegenüber der alleinigen ADT zeigen.

Eine Network-Metaanalyse bietet die Möglichkeit eines Rankings der einzelnen Substanzen

Das statistische Modell der Network-Metaanalyse bietet über dem hinaus noch die Möglichkeit eines sog. Rankings der einzelnen Substanzen. Hierbei zeigte sich in der Analyse von Mori et al. [9] Abirateron in der Auswertung bezüglich des Gesamtkollektivs unabhängig des Metastasierungsmusters als beste Doublet-Kombinationstherapie, dicht gefolgt von Apalutamid und Enzalutamid. In dieser Analyse, wie auch in bereits anderen Analysen zeigt sich die Docetaxel-Chemotherapie als Kombinationstherapie mit dem geringsten Effekt auf das OS, als auch das PFS [10].

Die oben genannten Resultate des Vorteils der Doublet- im Vergleich zur alleinigen ADT-Therapie dürften keinen Urologen bzw. Urologin mehr überraschen und viel interessanter erscheint hinsichtlich dieser Erkenntnisse tatsächlich der indirekte Vergleich bezüglich der ARTA-Kombinationstherapien gegenüber der Docetaxel-Kombinationstherapie: Leider ergibt sich hierbei bislang in der Literatur noch kein so eindeutiges Bild. Die oben zitierte Network-Metaanalyse von Mori et al. [9] kommt zu dem Schluss eines signifikanten PFS-Vorteils der ARTA gegenüber der Docetaxel-Chemotherapie, wohingegen hier keine Daten zum OS genannt werden, dies allerdings auch als Proxy genutzt werden könnte, [11]. Ähnlich zeigt sich in der von unserer Arbeitsgruppe publizierten Network-Metaanalyse ein signifikanter OS-Nachteil der Kombinationstherapie von Docetaxel im Vergleich zu Doublet-Therapie mit ARTA [12]. Die möglichen Unterschiede in den verschiedenen Metaanalysen entstehen hierbei vor allem durch immer wieder neue Update-Publikationen der entsprechenden Studien, welche neue HR nach längeren Follow-up publizieren. Durch diese „reiferen“ Daten ergeben sich immer wieder neue, z. T. konkurrierende, Ergebnisse.

### Indirekter Vergleich der Doublet- vs. Triplet-Kombinationstherapie

Nun kann man eben jene gemachten Annahmen auch unter Einbeziehung der neuen Triplet-Therapien mit den Daten der PEACE-1- (Abirateron + Docetaxel + ADT vs. Docetaxel + ADT) und ARASENS-Studie (Darolutamid + Docetaxel + ADT vs. Docetaxel + ADT) machen. Hierbei ist zu beachten, dass in der ARASENS-Studie alle mHSPC eingeschlossen werden könnten, wohingegen bei der PEACE-1-Studie lediglich primär metastasierte mHSPC-Patienten inkludiert wurden (Tab. 1). Ebenso bestand die PEACE-1-Studie aus einem 2 × 2-Design, da Patienten zudem eine Radiatio des Primärtumors erhalten konnten [7, 8]. Vergleichend mit dem SOC der jeweiligen Studien zeigte die jeweilige Triplet-Therapie einen signifikanten OS-Vorteil gegenüber einer Kombinationstherapie mittels Docetaxel-Chemotherapie und ADT nach 36 respektive 43 Monaten medianen Follow-up in den jeweiligen Publikationen (PEACE-1: HR 0,75; ARASENS: HR: 0,68) [7, 8]. Poolt man diese Daten nun auch mit denen der Doublet-Kombinationstherapien in einem indirekten Vergleich einer Network-Metaanalyse, bestätigt sich, der absolute OS-Vorteil der Triplet-Therapie gegenüber einer alleinigen ADT mit einer HR von 0,54 (ARASENS: Darolutamid + Docetaxel + ADT) und 0,60 (PEACE-1: Abirateron + Docetaxel + ADT). Allerdings zeigt sich, dass der relative Vorteil der Triplet-Therapie eng gefolgt von den ARTA-Doublet-Kombinationstherapien aus Abirateron (HR: 0,64), Apalutamid (HR: 0,65) und Enzalutamid (HR: 0,66) und erst dann von der Docetaxel-Chemotherapie (HR: 0,80) ist [12].

Um den relativ geringen OS-Vorteil der Triplet-Therapie in der Gesamtkohorte weiter zu analysieren, sollten wir nun noch tiefer in die Materie eintauchen und eine Unterscheidung zwischen der sog. High- und Low-volume-Metastasierung nach CHAARTED vornehmen, da heutzutage niemand seine Patienten mehr unabhängig vom Metastasierungsmuster behandeln sollte.

Grundsätzlich bleibt anzumerken, dass ein indirekter Vergleich der Network-Metaanalysen immer mit Limitationen verbunden ist, da für die individuellen Unterschiede zwischen den einzelnen Studien statistisch nicht adjustiert werden kann. Es muss dem Leser/der Leserin also immer im Hinterkopf bleiben, dass sowohl beispielsweise die Metastasierungsmuster, der PSA bei Therapiebeginn, der Anteil der vorherigen Lokaltherapien oder der mediane Follow-up-Zeitraum und auch der Studienzeitraum einen maßgeblichen Einfluss auf die publizierten Überlebens‑, PFS-Zeiten als auch errechneten HR – sowohl in den initialen Publikationen, als auch dem indirekten Vergleich – ausüben (Tab. 1).

### High-volume-Metastasierung

Die sog. High-volume-Metastasierung, welche sich international in der Klassifikation der Metastasenlast durchgesetzt hat, basiert auf der Einteilung der CHAARTED-Studie: Das Vorliegen mindestens 4 ossärer Metastasen, wovon sich eine außerhalb des Achsenskeletts befinden muss, oder eine viszerale Metastase werden als „high volume“ klassifiziert [2, 13]. Beachtenswert hierbei ist, dass diese Klassifikation lediglich auf einem konventionellen Staging fußt (Skelettszintigraphie und CT). Grundsätzlich ist im klinischen Alltag eine deutliche Zunahme der High-volume-Metastasierung durch den flächendeckenden vermehrten Einsatz des PSMA-PET/CT (prostataspezifisches Membranantigen – Positronenemissionstomographie/Computertomographie) als Staginguntersuchung zu erwarten. Wie sich dies mit den Zulassungen der medikamentösen Therapie beim mHSPC sowie dem Tumoransprechen von vormals nicht detektablen kleineren Metastasen bzgl. der Endpunkte wie dem OS und PFS auswirken wird, bleibt allerdings abzuwarten und spekulativ.

Die High-volume-Metastasierung basiert auf der Einteilung der CHAARTED-Studie

Grundsätzlich hat sich bis zur Veröffentlichung der Triplet-Therapieergebnisse der Urologe/die Urologin bei der Behandlung des High-volume-mHSPC-Patienten die Frage gestellt, startet man in der Erstlinientherapie mit ADT + ARTA oder ADT + Docetaxel? Zwar gab es bereits Hinweise, dass die Doublet-Therapie aus ADT + ARTA ein besseres Therapieansprechen haben könnte, doch beide Therapieoptionen wurden stets als noch äquivalent angesehen. Beispielsweise zeigte sich bereits in einer gepoolten Metaanalyse aller verfügbaren Phase-III-Studien bzgl. Doublet-Therapien von Docetaxel + ADT und Abirateron + ADT, dass im Setting der High-volume-Metastasierung das mediane OS mit Abirateron + ADT bei 50,1 Monaten vs. 45,9 Monaten für Docetaxel + ADT liegt [14].

Mit den nun vorliegenden Daten der PEACE-1-Studie hat sich dieses Konzept allerdings grundlegend verändert [7]: Die Triplet-Therapie aus Abirateron + Docetaxel + ADT zeigte im Setting der High-volume-Metastasierung einen signifikanten Überlebensvorteil im Vergleich zur Doublet-Therapie aus Docetaxel + ADT mit einer HR von 0,72 sowie einer ebenso signifikant längerem PFS (HR 0,47). Übersetzt in absolute Zahlen bedeutet dies ein medianes OS von 5,1 vs. 3,5 Jahren sowie ein medianes PFS von 4,1 vs. 1,6 Jahren zugunsten der Triplet-Therapie. Erwähnenswert und wichtig in der Interpretation dieser Daten bleibt allerdings auch, dass einige mHSPC-Patienten in diesem Studiensetting zudem eine lokale Radiotherapie erhalten haben, was wohl mit Sicherheit einen Einfluss auf die Länge der oben genannten Endpunkte gehabt haben dürfte, allerdings keinen Unterschied in der Interpretation des grundsätzlichen Vorteils der Triplet- gegenüber der Doublet-Therapie darstellt. Aus diesen Daten dürfte beim High-volume-mHSPC die Triplet-Therapie die Doublet-Therapie aus ADT + Docetaxel obligat gemacht haben. Leider wurden bis zum heutigen Stand keine Daten bzgl. der Triplet-Therapie der ARASENS-Studie bestehend Darolutamid + Docetaxel + ADT im Setting der High-volume-Metastasierung veröffentlicht [8].

Aus rein wissenschaftlicher Betrachtungsweise der Triple-Therapie wäre natürliche ein Studiendesign aus ADT + ARTA (SOC) ± Docetaxel wünschenswert, um den additiven Benefit der Chemotherapie im Setting des mHSPC zu evaluieren. Leider ist es unrealistisch, dass diese Art Studie initiiert und durchgeführt wird. Um einen etwaigen Proxy für diese Art von Analyse beim High-volume-mHSPC zu erhalten, bedarf es erneut einen Blick in den indirekten Vergleich im Rahmen einer Network-Metaanalyse. Hier konnte unsere Arbeitsgruppe nach Pooling aller verfügbaren Doublet- und Triplet-Therapien zeigen, dass die Triplet-Therapie aus ADT + Abirateron + Docetaxel dem der Doublet-Therapie mit den besten OS-Outcomes (ADT + Abirateron) beim High-volume-mHSPC überlegen sein könnte (HR für das OS 0,52 vs. 0,61; [12]). Zu nahezu selben Schlüssen kommen methodisch ähnliche Reviews und Network-Metaanalysen anderer Arbeitsgruppen [15–18].

### Low-volume-Metastasierung

Das Stadium des Low-volume-mHSPC klassifiziert ein sehr heterogenes Patientenkollektiv. Von singulär oder oligoossär metastasierten Patienten, die sich einer Lokaltherapie der Prostata unterziehen, bis hin zu Patienten mit vollständig durchmetastasierter Wirbelsäule fallen all jene mHSPC-Patienten in diese Kategorie. Hinsichtlich der Daten der Doublet-Therapiestudien zeigte sich, dass eine Kombinationstherapie aus ADT + Enzalutamid oder Apalutamid eine Verlängerung des OS bewirkt, was eine Zulassung in diesem Setting bewirkte [4, 6]. Schaut man sich die Daten zu Abirateron an, zeigen sich unterschiedliche Ergebnisse der Phase-III-Studien: Während sich in der LATITUDE-Studie für Low-risk-mHSPC-Patienten (als Proxy für „low volume“) keinen OS-Vorteil zeigte, zeigte sich im STAMPEDE-G-Arm ein OS-Vorteil mit Abirateron auch im Low-volume-mHSPC [3, 19]. Die Zulassung von Abirateron beschränkt sich somit nur auf das High-risk-Stadium nach LATITUDE. Der Benefit einer Docetaxel-Chemotherapie beim Low-volume-mHSPC wird seit jeher uneinheitlich diskutiert [2, 20].

Die Triplet-Therapie hat noch keinen Stellenwert bei Low-volume-mHSPC

Interessant war nun, wie sich die Triplet-Kombination aus ADT + Docetaxel + Abirateron auf das OS im Low-volume-mHSPC auswirkt: Aber auch hier zeigen die neueren Daten aus der PEACE-1-Studie im Setting des Low-volume-mHSPC keinen signifikanten OS-Benefit, wenngleich einen signifikanten PFS-Vorteil zugunsten der Triplet-Therapie (HR: 0,58, medianes PFS: Nicht erreicht vs. 2,7 Jahre) [7]. Wie auch beim High-volume-mHSPC, liegen auch beim Low-volume-mHSPC aktuell noch keine Daten zur Triplet-Therapie aus der ARASENS-Studie vor, werden aber für den kommenden ASCO-GU 2023 erwartet. Die oben genannten Überlegungen müssen allerdings beim Low-volume-mHSPC immer unter Berücksichtigung eines in den Studien kleineren Patientenkollektivs, längeres benötigtes Follow-up bis zum „event of interest“ (Tod) aufgrund der besseren Prognose getroffen werden. Entsprechend lässt sich zusammenfassen, dass die Triplet-Therapie aktuell (noch) keinen Stellenwert in der Behandlung des unselektierten inhomogenen Kollektivs des Low-volume-mHSPC darstellt.

## Nebenwirkungspotential

Die Nebenwirkungen unter ARTA-Therapien beim mHSPC sind nur in seltensten Fällen sehr ausgeprägt und beziehen sich am häufigsten auf das Herz-Kreislauf-System (Hypertension, Flush) oder Elektrolytstörungen (Abirateron). Demgegenüber ist die Docetaxel-Chemotherapie deutlich nebenwirkungsreicher mit den typischen chemotherapieassoziierten Nebenwirkungen wie Anämie, Thrombozytopenie, Neutropenie, Thrombose oder Fieber/Sepsis. Entsprechend interessant waren die ersten Daten hinsichtlich der Triplet-Therapien mit den Kombinationen aus Chemotherapie und ARTA, v. a. in Anbetracht, dass Darolutamid aufgrund seiner hydrophilen Seitenkettenstruktur durch eine geringere Penetration der Blut-Hirn-Schranke ein geringes (v. a. des zentralnervösen) Nebenwirkungsprofil zu haben scheint [21]: Im PEACE-1-Trial zunächst zeigte sich eine 11 % höhere Rate an aller Grad ≥ 3 „adverse events“ (AE) zu Ungunsten der Triplet-Therapie bestehend aus ADT + Abirateron + Docetaxel im Vergleich zur Doublet-Therapie mit ADT + Docetaxel (63 vs. 52 %; [7]). Dieser Unterschied fußte hier v. a. auf der Differenz der neu aufgetretenen Grad ≥ 3 Hypertension in der Gruppe mit zusätzlich Abirateron zur Chemotherapie (22 vs. 13 %). Diese Daten erscheinen konsistent mit denen der Abirateron-mHSPC-Zulassungsstudie LATITUDE, in denen sich auch eine 20 %-Rate an Hypertension Grad ≥ 3 zeigte [3]. Andere Grad ≥ 3-AE zeigten sich im Vergleich der Triplet- und Doublet-Therapie als nicht klinisch relevant unterschiedlich und jeweils ≤ 10 % der Fälle auftretend. Grad-5-AE, entsprechend eines tödlichen Verlaufs, traten hier in 2 bzw. 1 % der Triplet- bzw. Doublet-Therapie auf. Beachtenswert zeigte sich demgegenüber allerdings in der ARASENS-Studie eine relativ hohe Rate von 4 % Grad-5-AE in der Triplet- als auch Doublet-Therapiegruppe (und kann damit am ehesten Docetaxel-assoziiert interpretiert werden; [8]). Ebenso zeigten sich darüber hinaus hohe Raten an Grad-3- bis -4-AE von 66,1 und 63,5 %, welche zu je einem Drittel auf eine Neutropenie in sowohl der Triplet- als auch Doublet-Therapie zurückzuführen war (Vergleich Neutropenie Grad-3- bis -4-AE in PEACE-1-Studie: 10 bzw. 9 %). Die Hypertension zeigt auch hier in der ARASENS-Studie eine der häufigsten Nebenwirkungen v. a. in der Triplet-Therapiegruppe, allerdings mit deutlich geringer Inzidenz als in der PEACE-1-Studie mit 6,4 vs. 3,2 %. Insgesamt bleibt jedoch anzumerken, dass es keine klinisch signifikanten Unterschiede über alle AE-Grade hinweg in der ARASENS-Studie zwischen den Vergleichsgruppen gab.

Zusammenfassend lässt sich sagen, dass es sowohl mit Doublet- als auch Tripletherapien in mindestens jedem zweiten Patienten aus den wohlselektierten Studienkollektiven zu Grad ≥ 3-Nebenwirkungen bei der Behandlung des mHSPC kommt. In der Realität dürften die Raten wohl noch höher liegen. Allerdings scheint die additive Triplet-Therapie die Nebenwirkungsrate nicht zu potenzieren. Das Gros, je nach Kombination der Triplet-Therapie mit Darolutamid oder Abirateron, ist hier auf die chemotherapieassoziierte Neutropenie oder die Hypertension runterzubrechen. Dieses Wissen sollte die Basis in der Behandlung mit den genannten Substanzen sein, ebenso die Gewissheit, dass bis zu jeder 25. Patient unter einer Chemotherapie im Rahmen der Doublet- oder Triplet-Therapie beim mHSPC an Grad-5-AE versterben kann.

## Ausblick und Praxisrelevantes

Die neuen Daten zur Triplet-Therapie haben mal wieder zu einem Umbruch in der Behandlung des mHSPC geführt. Nach der direkten, als auch indirekten Betrachtung aller momentan vorliegenden Daten beim mHSPC kann man davon sprechen, dass die ADT + ARTA unabhängig vom Metastasierungsmuster den „backbone“ in der mHSPC-Behandlung darstellt. Docetaxel sollte aufgrund der Unterlegenheit im Vergleich zur ARTA-Therapie nicht mehr primär beim mHSPC zum Einsatz kommen.

Docetaxel sollte nicht mehr primär beim mHSPC zum Einsatz kommen

Bei einem ausgewählten mHSPC-Patientenkollektiv – wobei dieses Kollektiv noch durch weitere Analyse von Subgruppen genauer definiert werden muss, aber Faktoren wie High-volume-Metastasierung, guter ECOG-Status, geringes Patientenalter einfließen werden – wird die Triplet-Therapie eine Möglichkeit darstellen, einen noch höheren Therapienutzen hinsichtlich des Gesamtüberlebens unter milder Erhöhung der Nebenwirkungsrate zu erzielen.

## Fazit für die Praxis

- Innerhalb weniger Jahre zeigten Kombinationstherapien einen signifikanten Überlebensvorteil mit einer Kombination aus Androgendeprivationstherapie (ADT) plus Docetaxel-Chemotherapie oder neueren Androgenrezeptorantagonisten („androgen receptor targeted agent“, ARTA) bei zunächst spezifischen Metastasierungsmustern.
- Das Feld der medikamentösen Behandlung des metastasierten hormonsensitiven Prostatakarzinoms (mHSPC) hat einen grundlegenden Wandel innerhalb der letzten 10 Jahre durchlebt.
- Es gibt im Stadium des mHSPC aktuell vier in Deutschland zugelassene Kombinationstherapien.
- Das statistische Modell der Network-Metaanalyse bietet über dem hinaus noch die Möglichkeit eines sog. Rankings der einzelnen Substanzen.
- Die Triplet-Therapie aus Abirateron + Docetaxel + ADT zeigte im Setting der High-volume-Metastasierung einen signifikanten Überlebensvorteil im Vergleich zur Doublet-Therapie aus Docetaxel + ADT.
- Docetaxel sollte nicht mehr primär beim mHSPC zum Einsatz kommen.
